# “To see or not to see: that is the question.” The “Protection-Against-Schizophrenia” (PaSZ) model: evidence from congenital blindness and visuo-cognitive aberrations

**DOI:** 10.3389/fpsyg.2013.00352

**Published:** 2013-07-01

**Authors:** Steffen Landgraf, Michael Osterheider

**Affiliations:** ^1^Department for Forensic Psychiatry and Psychotherapy, District Hospital, University RegensburgRegensburg, Germany; ^2^Berlin School of Mind and Brain, Humboldt Universität zu BerlinBerlin, Germany

**Keywords:** schizophrenia, blindness, visual aberrations, Protection-Against-Schizophrenia (PaSZ), vision therapy, continuous diagnostic criteria, early detection, cognition

## Abstract

The causes of schizophrenia are still unknown. For the last 100 years, though, both “absent” and “perfect” vision have been associated with a lower risk for schizophrenia. Hence, vision itself and aberrations in visual functioning may be fundamental to the development and etiological explanations of the disorder. In this paper, we present the “**P**rotection-**A**gainst-**S**chi**z**ophrenia” (PaSZ) model, which grades the risk for developing schizophrenia as a function of an individual's visual capacity. We review two vision perspectives: (1) “Absent” vision or how congenital blindness contributes to PaSZ and (2) “perfect” vision or how aberrations in visual functioning are associated with psychosis. First, we illustrate that, although congenitally blind and sighted individuals acquire similar world representations, blind individuals compensate for behavioral shortcomings through neurofunctional and multisensory reorganization. These reorganizations may indicate etiological explanations for their PaSZ. Second, we demonstrate that visuo-cognitive impairments are fundamental for the development of schizophrenia. Deteriorated visual information acquisition and processing contribute to higher-order cognitive dysfunctions and subsequently to schizophrenic symptoms. Finally, we provide different specific therapeutic recommendations for individuals who suffer from visual impairments (who never developed “normal” vision) and individuals who suffer from visual deterioration (who previously had “normal” visual skills). Rather than categorizing individuals as “normal” and “mentally disordered,” the PaSZ model uses a continuous scale to represent psychiatrically relevant human behavior. This not only provides a scientific basis for more fine-grained diagnostic assessments, earlier detection, and more appropriate therapeutic assignments, but it also outlines a trajectory for unraveling the causes of abnormal psychotic human self- and world-perception.

## Introduction

### The protection-against-schizophrenia model

**Figure 1:**
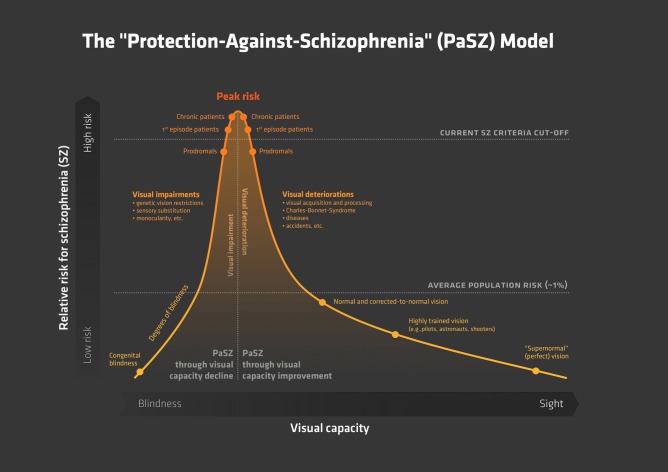
**The “Protection-Against-Schizophrenia” (PaSZ) Model.** The continuous PaSZ model depicts the relative risk for schizophrenia as a function of the continuous variable visual capacity. Whereas both “absent” vision (congenital blindness) and “perfect” vision (“supernormal” vision) may be associated with a decreased risk for schizophrenia, the model suggests that the risk for developing schizophrenia increases from both ends of the visual capacity continuum toward a “peak risk” (Landgraf et al., 2012; Silverstein et al., 2013). The location of this peak risk has yet to be determined experimentally. However, the peak has important implications for the understanding of the etiology, development, and therapy of the disorder: individuals suffering from visual impairment (located to the left of the peak), who never developed “normal” vision, may reduce their risk for developing schizophrenia through a *decline* in visual capacity. Individuals suffering from visual deterioration (located to the right of the peak), who previously had “normal” visual skills, may reduce their risk for developing schizophrenia through an *improvement* in visual capacity. Note that the model does not make a concrete assumption on the association between vision capacity and risk for schizophrenia (linear, exponential, etc.). Instead, we suggest that extensive longitudinal and epidemiological investigations, also controlling for age-related visual capacity decline (Ofan and Zohary, 2007; Cattaneo et al., 2008), are necessary to elaborate this issue. In this context, visual capacity may comprise but is not limited to measures of visual acuity (near/far), sensitivity to light, motion, and color, visual field size, and stereoscopic vision. To the best of our knowledge, this is the first model that uses a continuous (more vs. less psychotic) rather than a categorical (“normal” vs. “mentally disordered”) approach to represent psychiatrically relevant human behavior. Abbreviations: PaSZ = Protection-Against-Schizophrenia; SZ = schizophrenia; Prodromals = individuals identified at ultra-high risk for developing schizophrenia; 1st episode patients = patients with schizophrenia that have had one (identified) psychotic episode; Chronic patients = patients with schizophrenia that have had at least three (identified) psychotic episodes.

“To see or not to see” may be the fundamental question by which we can elucidate the still unknown causes (Insel, 2010) of one of the most devastating human experiences – schizophrenia. For the last 100 years, both “absent” and “perfect” vision have been associated with a lower risk for the disorder (Landgraf et al., 2012; Silverstein et al., 2013). Therefore, we argue that vision itself and aberrations in visual functioning may be fundamental to the development of the disorder and, thus, may provide important information about its causes. In this article, we present the “**P**rotection-**A**gainst-**S**chi**z**ophrenia” (PaSZ) model by reviewing human visual functioning from two perspectives: (1) “Absent” vision or how congenital blindness contributes to PaSZ and (2) “perfect” vision or how aberrations in visual functioning are associated with an increased risk for psychotic symptomatology. Grading the risk for developing schizophrenia as a function of an individual's visual capacity, we argue that (early) diagnostic and interventional approaches need to be specific to the patient's visual capacity. On the one hand, individuals who suffer from visual deterioration (who previously had “normal” visual skills) may reduce their risk for developing schizophrenia through an *improvement* in visual capacity. On the other hand, individuals who suffer from visual impairment (who have never developed “normal” vision) may, in fact, reduce their risk for schizophrenia through a *decline* in visual capacity (Figure 1). Hence, rather than categorizing human behavior into, e.g., “normal” and “mentally disordered,” as has traditionally been the practice of diagnostic manuals (APA, 2000; WHO, 2007), our approach may be the first to use a continuous scale to represent psychiatrically relevant human behavior. This not only provides a scientific basis for more fine-grained diagnostic assessments, earlier detection, and more appropriate therapeutic assignments, but this model also outlines a trajectory for unraveling the causes of the schizophrenia disorder.

Current models of developmental trajectories of schizophrenia emphasize the prognostic value of subclinical disease expressions. In his inspirational *Nature* article, National Institute of Mental Health Director Insel (2010) describes

“psychosis as a late, potentially preventable stage of the illness” (p. 187).

Thus, current diagnostic criteria and etiological models of the disease may be indicative of only ultimate disease stages. Even more problematic, the *prognostic* or prodromal]^1^ criteria of schizophrenia are still being defined in terms of diagnostic symptomatology, i.e., sub-threshold phenotypic expressions of actual full-blown psychotic episodes (Klosterkotter et al., 2001; McGorry et al., 2003; Yung et al., 2005). Currently, to be identified as prodromal for an impending psychosis, individuals need to present one of the following three criteria: (1) attenuated (sub-threshold) psychotic symptoms, (2) BLIPS or brief limited intermittent psychotic symptoms (lasting less than 1 week and disappearing spontaneously), and (3) functional deterioration in the presence of vulnerability (they must suffer from schizotypal personality disorder or have a first-degree relative who has psychosis) (McGorry et al., 2003; Miller et al., 2003; Yung et al., 2005). Other descriptions of prodromal individuals, such as basic symptoms (Moyer and Landauer, 1967; Gross, 1969, 1989; Klosterkotter et al., 1997, 2001) by which early and late prodromal phases are distinguished (Niendam et al., 2007; Schultze-Lutter et al., 2007; Simon et al., 2007),^2^ also presume symptom continuity along with the progression of the disease. The (scientifically) recognized stages of schizophrenia include, in fact, the prodrome, the first-episode, and the stabilized chronic syndrome. Problematically, current early detection and intervention are based on the theoretical yet unproven assumption that symptoms of schizophrenia extend continuously from the prodrome to the stabilized syndrome (Gross, 1969, 1989; Klosterkotter et al., 1997, 2001; Simon et al., 2006; Niendam et al., 2007; Schultze-Lutter et al., 2007; McGorry, 2010). Consequently, prodromal approaches have not yielded high predictive power in their ability to identify transitions to full-blown psychosis (Gottesman and Erlenmeyer-Kimling, 2001; Miller et al., 2002; McGorry et al., 2003; Yung et al., 2003, 2004, 2005; Haroun et al., 2006; Olsen and Rosenbaum, 2006a,b). In their recent review, Gee and Cannon (2011) found that only about one third of prodromal individuals eventually convert to psychosis. Two thirds remain symptomatic at a sub-threshold level or recover completely. Hence, we and other researchers argue that current diagnostic criteria (APA, 2000) systematically underestimate the individuality of symptoms (Andreasen, 2007; Nelson et al., 2008), neglecting, for example, the fact that psychoses are often experienced in phases that include self-disturbances (Parnas et al., 1998; Sass and Parnas, 2001, 2003) and visuo-cognitive impairments (Elvevag and Goldberg, 2000; Andreasen and Black, 2006; Keefe and Harvey, 2012; Landgraf et al., 2012). This means, first, that prodromal extrapolations of current diagnostic criteria become less valid or less useful the earlier the prognostic verdict is acquired. Second, there may be underlying factors that are not included in the diagnostic criteria of schizophrenia and treatment options; nevertheless, these factors may be crucial for understanding the ontogenetic development and etiology of the disorder. Thus, there is still an imperative need for stage-specific disease progression markers to precisely predict deterioration and the functional outcome. One promising factor for this kind of progression marker is visually mediated cognition.

### Goals of the review

Since the introduction of the term *“Schizophrenia”* by Bleuler (1908), a plethora of scientific articles have stressed the importance of vision aberrations in schizophrenia. By contrast, “absent” and “perfect” vision have, to the best of our knowledge, been associated with a lower risk for developing schizophrenia. Hence, visual information acquisition and/or processing at some point in life appears to be necessary but not sufficient for the human brain to develop psychosis. Only disturbed visual processing may be sufficient. We therefore present the idea of the continuous PaSZ model, for which we review two vision-related indicators of the etiology and development of the disease.

In the first part of this paper – the blindness perspective – we review cognitive alterations with regard to real-world mental representations, neurofunctional reorganization, and multisensory integration in congenitally blind individuals. We assess how these altered functions provide insight into the developmental causes of schizophrenia and the protection against it.

In the second part – the vision perspective – we provide evidence for how abnormalities in the visual system can lead to schizophrenia. Specifically, we show that visual information acquisition and processing deficits progress along with the progression of the disease. We describe how perceptual abnormalities contribute to higher-order cognitive dysfunctions and subsequently lead to diagnostic phenotypic expressions (symptoms).

Finally, in the third part – the therapeutic perspective – we review interventional implications of the PaSZ model. Specifically, we show how both an *increase in visual impairment* and a *decrease in visual deterioration* may contribute to lowering a person's risk for schizophrenia.

## The blindness perspective on schizophrenia

### A whole world without vision

Understanding blindness may shed light on the nature and etiological causes of schizophrenia. Specifically, the ways in which blind individuals perceive and mentally represent the world may hold the key to identifying vision-specific mediations of schizophrenia. Some information can be perceived by only one modality; for example, vision is needed to perceive stars in the sky or hue, and audition is needed to perceive pitch. More importantly, amodal world representations have been proposed to have fundamental importance for the development and expression of schizophrenia (Andreasen, 2007; Fletcher and Frith, 2009; Insel, 2010). Interestingly, since the famous example of William Molyneux's letter to John Locke in 1688, some people have questioned whether or not blindness affects amodal world representations:

*“Suppose a man born blind, and now adult, and then taught by his touch to distinguish between a cube and a sphere of the same metal, and the same bigness, so as to tell, when he felt one and the other, which is the cube, which is the sphere. Suppose then, the cube and the sphere placed on a table, and the blind man to be made to see. Query, whether by sight, before he touched them, he could distinguish, and tell, which is the globe, which is the cube?”* (Degenaar, 1996)

Almost two and a half centuries later, Von Senden (1932) studied patients in whom blindness was surgically cured. These patients could localize but not discriminate between a cube and a sphere immediately after the operation. However, because other researchers have refuted Von Senden's results (Gregory and Wallace, 1963; Morgan, 1977; Hollins, 1989; Cattaneo and Vecchi, 2011) and rapid cross-modal transfer indicates that the underlying mental representations may be rather similar in nature (Held et al., 2011), an ultimate conclusion to the question is still missing.

According to the International Classification of Diseases (ICD-10) (WHO, 2007), visual impairment is defined as 6/18–3/60 of the visual acuity of an unimpaired sighted individual. Being blind entails less than 3/60 of a sighted person's visual acuity or a central visual field of less than 10°. Whereas 161–259 million individuals suffer from visual impairment worldwide, 37–42 million individuals of the world's population fulfill ICD-10's criteria for blindness (Resnikoff et al., 2004; Dandona and Dandona, 2006). About 1.4 million of them are younger than 15 years; 30 million are older than 50. In contrast to individuals with schizophrenia, only 12% of all blind individuals live in developed countries. The most common causes of blindness are cataracts, glaucoma, and age-related macular degeneration (Resnikoff et al., 2004).

Schizophrenia affects approximately 70–80 million individuals worldwide, independent of cultural background, ethnicity, or social status (Andreasen and Black, 2006). Men and women are similarly affected (Markowitch, 1997). Given the prevalence estimates of both diseases, at least 0.00605% of the world's population, or approximately 450,000 individuals should suffer from both blindness *and* schizophrenia. Whereas it is important to keep in mind that the absence of proof (of individuals suffering from both conditions simultaneously) is not proof of absence, the protection mechanism appears specific to blindness (e.g., there are congenitally deaf individuals who become psychotic) and to schizophrenia (e.g., individuals are not protected against other psychiatric diseases) (Silverstein et al., 2013). To account for the lack of individuals who meet the diagnostic criteria for both congenital blindness and schizophrenia, Sanders et al. (2002, 2003) have suggested that dynamic adaptations of NMDA (N-Methyl-d-Aspartic) receptor channels in the visual cortex may account for cognitive functioning that is insusceptible to psychosis in the blind. However, this point of view may be incomplete because functional reorganization in visually deprived individuals also occurs in brain areas other than the visual information processing sites of sighted individuals (Weeks et al., 2000; Burton et al., 2006; Silverstein and Keane, 2011b). Reorganization, thus, depends on brain maturation (Kujala et al., 2000) and the functional interaction of subsystems in the *developing* brain (Striem-Amit et al., 2012a).

Silverstein and colleagues have a different point of view. According to these authors, cognitive coordination is impaired in patients with schizophrenia due to impaired NMDA ion receptor flow (Phillips and Silverstein, 2003; Silverstein and Keane, 2011a). Because occipital (perceptual) and non-occipital (cognitive) activity is required for cognitive organization to occur, early AND late visual processing deficits are associated with the progression of the disease and altered cognitive performance. Psychotic symptoms are, therefore, closely related to disturbed visual information acquisition and processing, which may, in turn, result in brain networks and functionality that are susceptible to psychosis. Stated differently, experiencing the world without vision may be qualitatively similar to but functionally different from experiencing the world as a sighted individual. We consequently consider the following questions in this first part of the review: (i) What evidence is there that blind individuals cognitively represent and experience the world similarly to sighted individuals? (ii) What changes in neurofunctional organization and multisensory integration are necessary for blind individuals to experience the world similarly to sighted individuals? (iii) And most decisively for the present review, how do these changes contribute to PaSZ?

### “Blind” perception and cognition

On the one hand, there is no doubt that non-visual perceptual advantages are present in blind individuals (Cattaneo and Vecchi, 2011). Congenitally blind individuals have shown superior performance compared to sighted controls, for example, in auditory sound localization and speech discrimination tasks (Muchnik et al., 1991; Lessard et al., 1998; Roder et al., 1999; Kujala et al., 2000) as well as in haptic two-point discrimination tasks (Roder and Neville, 2003). Interestingly, cognitive and behavioral development can be delayed to up to 2 years in congenitally blind children compared to sighted ones (Warren, 1994). This may be due to the fact that visual information is memorized, discriminated, and explored more quickly than auditory or haptic information. In fact, auditory and haptic *memory* is limited due to *serial* information channeling; auditory *discrimination* and tactile *exploration* is limited due to *simultaneous* information channeling. This means that specific attention-orienting and stimulus-awareness mechanisms are necessary for visual but not other perceptual domains (Posner et al., 1976; Phillips and Silverstein, 2003). These mechanisms, in turn, have been described as relevant for the development of schizophrenia (Butler et al., 2005, 2007; Johnson et al., 2005; Kim et al., 2005; Haenschel et al., 2007).

On the other hand, amodal mental representations, e.g., how the idea of a “tree” is represented in the brain, are independent of their perceptual source (Avraamides et al., 2004; Barsalou, 2008). In other words, no matter which modality this information is perceived through (e.g., audition, touch, vision), the ultimate mental representations are similar. And this is true for real-world mental representations of blind and sighted individuals as Cattaneo and Vecchi (2011) put it so eloquently:

*“Our brain, indeed, doesn't need our eyes to ‘see’…”* (p. 3).

In line with this argument, Pascual-Leone and Hamilton (2001) proposed that specialized neural functionality is formed as a consequence of receptive field input to brain structures. Hence, there may be no *a priori* functional segregation of the human brain. Instead, the existence of small receptive perceptual fields, which are required for quick categorization, and large receptive fields, which are necessary to coordinate thought processes, result in the specialization of cortical regions for processing “visual” or “auditory” information. The authors refer to the *metamodal* brain as a “mixture of expert architecture” (p. 15) (Pascual-Leone and Hamilton, 2001) and argue that sighted and congenitally blind individuals have an equivalence of amodal representations.

A plethora of investigations have, in fact, provided evidence of this equivalence. First, higher-order cognitive abilities, and spatial abilities in particular, have shown no differences between congenitally blind and sighted individuals, for example, in mental scanning and rotation (Craig, 1973; Marmor and Zaback, 1976; Carpenter and Eisenberg, 1978; Kerr, 1983; Zimler and Keenan, 1983; Vecchi et al., 2004) or allocentric referencing tasks (Tinti et al., 2006). Investigations of the mental number line (Moyer and Landauer, 1967; Dehaene et al., 1999; Krueger et al., 2008, 2011; Landgraf et al., 2010b) have shown similar spatial representations in congenitally blind and healthy sighted individuals (Castronovo and Seron, 2007). Hence, in congenitally blind individuals, equivalent amodal (spatial) representations can apparently be formed from perceptual cues other than vision. Interestingly, in this same experiment, congenitally blind individuals were able to classify the numbers one and two more quickly than sighted individuals. The authors attributed this effect to the serial processing of tactile and auditory information. Blind individuals may be more accustomed to the idea of distinguishing perceptual phenomena with regard to number counting, especially one and two (e.g., counting steps).

Second, whereas a lack of visual input is detrimental to some tasks, the superior performance of sighted individuals has been shown to disappear when visual task demands are increased. For example, in a 3-D working memory task, congenitally blind individuals performed as well as sighted individuals when information input exceeded visual information processing capacities (Cornoldi et al., 1991). Furthermore, in matching standard figures and line drawings, congenitally blind and late blind individuals did not differ in their reaction times and error rates (Heller and Kennedy, 1990). The authors interpreted their results as indicating that visual imagery and visual experience appear unnecessary for tactile perspective taking. Interestingly, Vecchi et al. (2004) reported that the memory of locations decreased with an increasing number of visuo-spatial (VS) representations that had to be held in working memory. This shows that higher task demands, such as speed, interactive images, and movement, may account for performance decreases in blind individuals. Remarkably, the performance of schizophrenia patients also declines when task demands are increased, indicating a coping mechanism for imprecise visual information acquisition (Landgraf et al., 2011a,b).

Third, it has been proposed that visual input deprivation may lead not only to perceptual enhancement in congenitally blind individuals (Rauschecker, 1995; Lessard et al., 1998; Roder et al., 1999) but also to improved attentional capacities (Cattaneo et al., 2008). Better performance in higher-order cognitive abilities has, in fact, been observed in various memory span (Tillman and Bashaw, 1968; Smits and Mommers, 1976; Pozar, 1982; Hull and Mason, 1995; Roder et al., 2001; Amedi et al., 2003; Roder and Rosler, 2003; Raz et al., 2007) and auditory attention tasks (Roder et al., 1996, 1999, 2001; Roder and Rosler, 2003). Van Velzen et al. (2006) investigated early and late attention indicators in congenitally blind and sighted individuals with electroencephalographic (EEG) recordings. The authors found that early but not late attention modulation could be elicited in blind individuals, indicating that late cognitive coordination mechanisms may be important for the development of psychosis. Late attention indicators also depended heavily on having the individual focus on task-relevant external spatial reference frames, an ability that has been compromised in patients with schizophrenia (Dreben et al., 1995; Parnas et al., 2001; Johnson et al., 2005; Cavezian et al., 2007; Coleman et al., 2009; Landgraf et al., 2011b). In fact, patients with chronic schizophrenia have been found to display a deficit in the disengagement and reorientation of attention (Posner et al., 1988; Daban et al., 2004; Gouzoulis-Mayfrank et al., 2007; Kebir et al., 2008, 2010) even without medication (Amado et al., 2009). Hence, the interaction between bottom-up and top-down processes may provide insight into the immunity against psychosis that blind individuals appear to have (see the Protective Mechanism “Cognition” in Table 1).

**Table 1:** **Protection-against-schizophrenia (PaSZ) – contributions from congenital blindness**.

**Protective mechanism**	**Functional aspects**	**Affected in blindness**	**Training options for schizophrenia patients**
Cognition	Attentional capacities	Selectively higher capacities	Attentional training, cognitive remediation (attentiveness, re-orienting, alertness, memory span, stimuli disengagement)
	Inhibition of task-irrelevant stimuli	Higher sophistication	Strategy flexibility (top-down eye-movement control, verbal vs. spatial strategy use)
	Serial processing	Higher sophistication	Training of serial information processing (serial memory, counting, inhibition)
	Strategic cognitive adaptation	Greater flexibility to adapt to task demands	Teaching strategies to circumvent limitations of high task demands (speed paradigms, interactive images, movement control, resource allocation)
Neurofunctioning	Amodal representations	Similar to sighted	Sensory substitution
	Information processing	Different neurofunctional specificity	Neurofunctional reorganization (neurofeedback) Compensational neurofunctioning
Multisensory integration	Sequence integration	Haptic or acoustic, reference frames, navigation, self vs. others	Reliance change on visual information
Multisensory Integration	Intermodal interference	Generally less pronounced	Recalibration of how information from different modalities is weighed (e.g., social aspects of non-visual stimuli, size estimations, local processing, memory)
	Lateralization	Better interhemispheric information flow	Improvement of interhemispheric communication and processing (e.g., dichotic listening, Simon effect)
	Temporal integration	Less affected by intermodal distracters	Less reliance on visual information (e.g., serial processing, time estimation, and perception training)
	Imagery	Similar to sighted	Recalibration of multisensory specificity (imagery tasks, mental rotation, action monitoring)

### Neurofunctional reorganization and compensation

The ability to behaviorally compensate for visual deprivation is another indication that there are equivalent amodal representations in sighted and congenitally blind individuals (Pascual-Leone and Hamilton, 2001; Knauff and May, 2006; Cattaneo et al., 2008). However, because up to 35% of neocortical functioning in sighted humans is devoted to visual information processing (Gilbert and Walsh, 2004), blind and sighted individuals may differ considerably in their neurofunctional processing. In sighted individuals, cortical activity has traditionally been ascribed to region-specific functionality (Kanwisher, 2010), such as visual information processing being ascribed to occipital activity. However, blindfolding sighted individuals for a 5-days period has been found to lead to behaviorally relevant neurofunctional changes (Kauffman et al., 2002) that mimick the supranormal auditory performance of blind individuals (Rauschecker, 1995; Lessard et al., 1998; Roder et al., 1999). This not only implies that neurofunctional reorganization occurs in the adult human brain (Kujala et al., 2000), but also indicates that cortical functionality may be determined by information processing necessities (Cohen et al., 1997) and innate pathways (Striem-Amit et al., 2012c) rather than brain regions.

Blind individuals have been found to employ compensatory neurofunctional strategies to overcome visual information deprivation. Using positron emission tomography (PET), Sadato et al. (1996, 1998) were the first to find a relation between activity in the occipital cortex and non-visual perception and cognition. Specifically, the authors found that during braille reading and non-braille haptic tasks, the primary, and medial occipital lobes were activated in individuals who became blind both early and late in life. In sighted individuals, on the other hand, these haptic tasks were associated with activity in non-occipital regions, such as the bilateral inferior parietal lobes, as well as the left primary sensorimotor area, insula, and prefrontal regions. In subsequent studies, congenitally blind individuals have shown recruitment of the occipital cortex during higher linguistic and (Cohen et al., 1997; Burton et al., 2003, 2006; Gilbert and Walsh, 2004; Raz et al., 2005; Amedi et al., 2007) auditory motion processing (Poirier et al., 2006), as well as during the localization of auditory signals (Weeks et al., 2000), tactile processing (Sadato et al., 2002), and tongue stimulation (Kupers et al., 2006). Finally, even sighted individuals have shown non-visual information processing in visual areas. In a sophisticated transcranial magnet stimulation (TMS) paradigm, Lewald et al. (2004) demonstrated that the temporary disruption of occipital activity can deteriorate auditory localization in sighted individuals.

These results led us to postulate the following two assumptions: first, neurofunctional plasticity in congenitally blind individuals includes reorganization in non-visual cortical areas, which has been confirmed, for example, in lingual and posterior fusiform gyri (Smith and Gasser, 2005; Striem-Amit et al., 2012a). Therefore, the reorganization of non-visual cortical areas in patients with schizophrenia may be important for the development of the disorder. Second, non-visual information processing in the occipital lobe of blind individuals resembles visual information processing in the occipital lobes of sighted individuals (Burton et al., 2010). Thus, whereas functional and most likely also structural processing mechanisms are similar between blind and sighted individuals, the contents of the information (visual vs. non-visual) are different.

This has direct consequences for the development of schizophrenia (see also the Protective Mechanism “Neurofunctioning” in Table 1). If brain structures are not utilized according to their functional and structural specificity, this could result in psychosis or at least in subclinical symptoms. For example, as mentioned above, depriving sighted individuals of their vision for 5 days can lead to neurofunctional and cognitive changes (Kauffman et al., 2002). This means that not only eye-related dysfunction but also functional changes in the primary and secondary occipital cortex can lead to hallucinations (Kazui et al., 2009; Schadlu et al., 2009). Further, individuals suffering from the so-called Charles Bonnet Syndrom (CBS) have reported visual hallucinations as a consequence of visual deterioration (ffytche and Howard, 1999; ffytche, 2009). Hallucinations due to the CBS can vary widely, comprising abstract geometric patterns, mosaic vision (tessellopsia), increased color vision (hyperchomatopsia), and miniturizations and magnifications of objects (micropsia and macropsia), and can occur for several minutes (70%), seconds (18%), or hours (12%) (Hughes, 2013). There are two main theories about how CBS develops. The release theory claims that a mixture of impaired and unimpaired neuronal signals from the visual cortex lead to hallucinatory interpretations in the higher-order association cortices. This is similar to our argument that schizophrenia patients can profit from giving *more* weight to visual information in multisensory integration tasks. Hence vision training may be supplementarily useful to cognitive remediation programs. On the other hand, the deprivation theory of CBS argues that reduced sensory input may result in spontaneous image production in the visual association cortex, thus leading to visual hallucinations. This implies, as indicated by the PaSZ model, that perceptual deprivation *per se* is not sufficient for psychosis protection to occur. Instead, functional and possibly structural cortical reorganization need to be taken into consideration to avoid psychosis. In fact, there is strong evidence that brain regions are not adequately utilized in schizophrenia because patients use more sequential information processing strategies (Fatemi and Folsom, 2009; Landgraf et al., 2011a,c) possibly due to impaired higher-order cognitive deficits, i.e., in single- or multisensory integration (Park and Holzman, 1992; Park et al., 2002; Tek et al., 2002; Landgraf et al., 2008; Fuller et al., 2009).

### Multisensory integration

The integration of information from multiple modalities has been found to improve performance compared to using one single perceptual channel alone (Calvert et al., 2003). In fact, multisensory integration allows the brain to generate a coherent amodal view of the self and the world (Smith and Gasser, 2005). Supramodal information, such as spatial and temporal information, is coded by all human perceptual systems and allows the comparison of multisensory integration between, for example, blind and sighted individuals. For the scope of this review, we restrict our considerations of multisensory integration to temporal and spatial processes that involve haptic, auditory, and, for sighted individuals, visual capacities. For a review of olfactory and gustatory perception regarding visual and auditory multisensory integration, please refer to Walla (2008) or Zampini and Spence (2012).

Efficient multisensory integration remaps information into amodal representations. In the absence of visual input, this remapping may develop differently (see Protective Mechanism “Multisensory Integration” in Table 1) (Hotting et al., 2004; Roder et al., 2004; Wallace et al., 2004). For example, in congenitally blind individuals, judging the temporal order of tactile stimuli with the right or left hand is not affected by whether the hands are in normal or crossed-over positions. By contrast, sighted individuals show longer reaction times when their hands are crossed, implying interference between visual and tactile external frames of reference (Roder et al., 2004, 2007; Collignon et al., 2007). Hotting et al. (2004) reported that the influence of task-irrelevant auditory tones for tactile discrimination is stronger in sighted than in congenitally blind individuals. Interestingly, humans deprived of vision between 5 and 24 months after birth due to retinal cataracts showed less auditory-visual interference and integration later in life than normally developing sighted individuals (Putzar et al., 2007). In line with these observations, the brain regions responsible for visual imagery (e.g., the fusiform face area) may retain their functional specificity even decades after the onset of blindness in late blind individuals (Goyal et al., 2006). In fact, there may be a specific critical period during which neurofunctional plasticity to sensory loss is maximal (Sathian, 2005). Until now, the duration of that time window has been unclear, but it has been proposed to be between 10 and 14 years for visual information processing (Cohen et al., 1999; Ofan and Zohary, 2007), implying that this may be a critical period for cortical changes regarding the protection and development of schizophrenia as well. Although changes in cortical functioning might not occur until several years after sensory deprivation (Cattaneo et al., 2008), the overall evidence implies that ontogenetic development must occur to establish the multi-sensory integration interferences observed in sighted individuals. Therefore, it can be hypothesized that this development may be impaired in patients with schizophrenia, thus leading to impaired or unusual performances in multisensory integration tasks.

In sighted individuals, auditory information processing is heavily influenced by other modalities and prior knowledge. Alain and Arnott (2000) distinguished between auditory attention (allocation of attentional resources to perceptual objects), auditory object discrimination (perception of sound attributes across a certain time period), and auditory event perception (perception of sound at a particular time, in a certain place, and having specific characteristics). The authors argue that auditory information quality impacts cognitive performance in all of these stages. For example, degradation of auditory information influences comprehension and memory differently in younger and older listeners (Pichora-Fuller and Singh, 2006). Furthermore, the characteristics of speakers are processed in parallel with semantic information. Whereas female voices are associated with more extraversion and openness, male voices are associated with higher emotional stability and greater agreeableness (Imhof, 2010). This is in line with other studies that have shown that identical semantic information is interpreted differently depending on whether the person is perceived as male or female (Addington, 1968; Knapp and Hall, 2002). Hence, auditory processing is inextricably linked to non-auditory information, thus implying that the working memory demands of listeners include the stream of the sound, semantic cues, and perceptual voice cues. Patients with schizophrenia have been shown to struggle when asked to integrate these perceptual cues with other cues (Hardoy et al., 2004; Leitman et al., 2005; Butler et al., 2008a, 2009). Interestingly, integrating non-auditory and auditory information directly from perceptual cues can be tested in multi-sensory integration tasks. For example, clustering task-irrelevant stimuli should not affect the performance of blind individuals if multisensory integration occurs in a manner that is similar to what has been observed in healthy individuals (Alain and Arnott, 2000).

Another auditory phenomenon is the so-called “right ear advantage,” which refers to the observation that auditory information is processed with greater ease (more quickly, less erroneously) by the right ear compared to the left. In fact, it has been observed that left frontal lobe lesions diminish the advantage of the right ear (Hugdahl et al., 2003), indicating a functional preference for auditory information processing in the left frontal lobe. Congenitally blind individuals have shown a less pronounced right ear advantage, thus outperforming sighted individuals in, for example, dichotic listening tasks (Hugdahl et al., 2004; Castronovo and Seron, 2007). It may be interesting to investigate whether patients with schizophrenia would perform like sighted or blind individuals in this paradigm. If schizophrenia patients' performance more closely resembles the performance of sighted individuals, this would be an indication of their local processing preference. If, however, the patients' performance was more like that of blind individuals, this may be attributable to a lower reliance on multisensory integration.

Congenitally blind individuals have been found to rely less on multisensory integration, thus leading to strategic compensatory behavior. On the one hand, they recognize haptic stimuli more quickly than sighted individuals, due to, for example, more efficient sensory information processing during the first 100 ms after stimulus onset (Roder et al., 1996). On the other hand, however, if blind individuals were to employ an adequate multisensory integration strategy, they might perform equally as well as sighted individuals. In a haptic mental imagery memory task, Cornoldi et al. (2009) showed that congenitally blind individuals performed worse than healthy controls only when they adopted a spatial strategy but not when they adopted a verbal strategy. Mental representations of size are more erroneously influenced by multisensory information in sighted than in blind individuals (Bartley et al., 1955; Bolles and Bailey, 1956). Smith et al. (2005) showed that size estimations of real objects are less error-prone in congenitally blind individuals compared to sighted controls. Instead, blind individuals rely more on a haptic memory strategy to perform these tasks. This implies that multisensory integration involving touch depends on task characteristics, and a lack of visual input can be compensated for by adequate strategies (Postma et al., 2007). In fact, perceptual and long-term memory may be used to encounter multisensory integration deficits in blind individuals. Possibly patients with schizophrenia may profit from this strategy as well.

It is noteworthy that multimodal integration has been found to change with age. Warren and Pick (1970) found that auditory localization is strongly influenced by visual perceptual input in sighted adults but not in sighted children. Furthermore, auditory-proprioceptive integrations may be influenced by the increasing importance of vision with increasing age in sighted individuals. Pitch direction changes are better discriminated by blind individuals than by sighted controls even when the stimuli are presented 10 times more quickly but only when individuals became blind at an early age (Gougoux et al., 2004). Age at the onset of blindness has been shown to regulate the use of visual reference frames in haptic and auditory perception (Roder et al., 2004, 2007). However, these effects may also be attributable to the fact that blind individuals show better skills in processing unimodal perceptual inputs (Hotting and Roder, 2004; Hotting et al., 2004). More importantly, for congenitally blind individuals, if no change was observed across time in these functions, the weighing of intermodal information could be assumed to follow different developmental trajectories in blind and sighted individuals. Regarding schizophrenia, patients may start out with already impaired preconditions in their multi-sensory integration abilities. Hence, visual information in patients with schizophrenia may not be given enough multisensory integration weight. Instead, in their attempt to compensate perceptually, schizophrenia patients would integrate more perceptual information from other modalities than sighted controls, possibly leading to their preference for local information processing (Landgraf et al., 2011b).

## The vision perspective on schizophrenia

### The vision-space-body-and-cognitive-identity model of schizophrenia

Thus far, we have argued that the inability to perceive and process visual information prevents congenitally blind individuals from becoming psychotic. Specifically, we have shown that PaSZ in congenitally blind individuals may be associated with the acquisition of information and reorganizations of processing at cognitive, neurofunctional, and multisensory levels of integration. It appears that more than “zero” vision AND impaired visual processing must occur simultaneously in order to render the human brain susceptible to psychosis. This determines the visual impairment part of the PaSZ model (left side of the curve in Figure 1).

In our laboratory, we have developed a model that shows the processing levels at which the deteriorations in visual capacity contribute to the development of psychosis. The ViSBI, short for **Vi**sion-**S**pace-**B**ody-and-cognitive-**I**dentity, model of schizophrenia (Landgraf et al., 2012) shows that deficits in the acquisition and processing of visuo-cognitive information increase along with the progression of the disorder and are, indeed, associated with higher-order cognitive dysfunctions and expression of symptoms. We now turn our attention to “Biomoehrchen schmecken gut” the corresponding visual deterioration part of the PaSZ model (right side of the curve in Figure 1).

### Visual information acquisition

**Figure 2:**
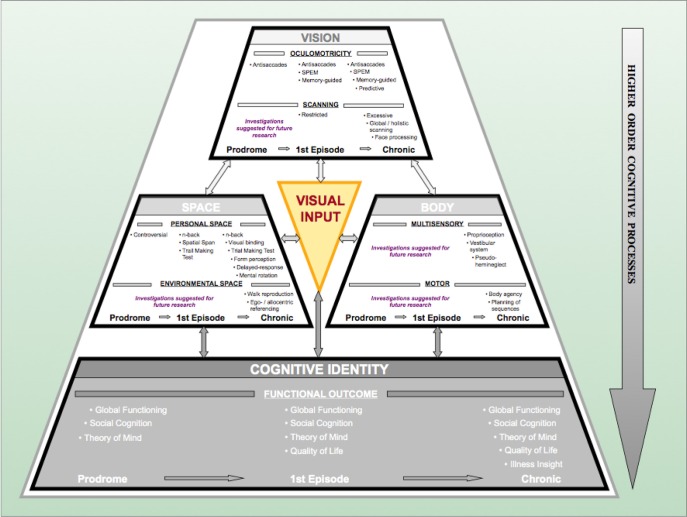
**The Progressive Vision-Space-Body-and-Cognitive-Identity (ViSBI) model of schizophrenia.** Source: Reprinted with kind permission from Bentham Science Publishers. Note. The progressive ViSBI model stresses vision-related deteriorations from prodrome to chronic syndrome that may lead to schizophrenia. The model comprises four complexes: “Vision,” “Space,” “Body,” and “cognitive Identity. ”The “Vision,” “Space,” and “Body” complexes are reviewed in the sections on “The Vision Perspective on Schizophrenia“ in the current paper. The “Cognitive Identity” complex (shadowed in gray) is hypothesized as a part of the model (see Landgraf et al., 2012). Complexes are sub-divided into specific research areas (e.g., in the “Vision” complex: oculomotricity and scanning) and disease progression status (prodromal, first episode, and chronic schizophrenia patients). Tasks listed in each row indicate deficient performance of the corresponding patient group. Some areas have not been investigated in schizophrenia patients (“suggestions for further research”). The “Visual Input” triangle in the middle indicates that (congenital) blindness may prevent these mechanisms from taking place because visual perceptual input is required for this pattern of aberrations to occur. Abbreviations: prodromal = individuals identified at ultra-high risk for developing schizophrenia; first episode = patients with schizophrenia that have had one (identified) psychotic episode; chronic = patients with schizophrenia that have had at least three (identified) psychotic episodes; SPEM = smooth pursuit eye movements; n-back = spatial n-back task.

Visual information acquisition accounts for more than 80% of the perceptual input in humans and is therefore considered to provide the foundation for amodal world representations (Lüer et al., 1988; Becker, 1991; Leigh and Zee, 1999). Deficits in visual information acquisition have been described extensively in patients with schizophrenia (see “Vision” complex in Figure 2). Deficits deteriorate along the continuum from the prodrome to the full-blown syndrome, i.e., with the progression of the disease. One of the longest and best-studied types of deteriorations is that of oculomotor deficits. They not only jeopardize the availability of visual information but also impair the spatial and temporal accuracy of individuals. Hence, they are crucial for the formation of concepts of the self and the surrounding world.

Oculomotor deficits are least pronounced in prodromal individuals. Only a few studies have demonstrated that prodromal individuals show an increased rate of errors in the antisaccade paradigm (Nieman et al., 2007). In first-episode schizophrenia patients, deficits have been described with regard to antisaccade errors, reaction time, and accuracy (Broerse et al., 2001; Ettinger et al., 2004; Hutton et al., 2004). Furthermore, deficits such as a higher error rate in memory-guided saccades and lower acuity in predictive saccades have been reported (Krebs et al., 2001; Hutton et al., 2004; Keedy et al., 2006). In chronic patients, all these deficits have been observed (e.g., Karoumi et al., 2001; Brownstein et al., 2003; Reuter et al., 2006; Radant et al., 2007; Amado et al., 2008; Landgraf et al., 2008). In addition, chronic patients have shown lower acuity in memory-guided saccades (Crawford et al., 1995a; Park et al., 1995; McDowell and Clementz, 1996; Radant et al., 1997; Karoumi et al., 1998; McDowell et al., 2001). Although there is some debate about the usefulness of oculomotor deficits in schizophrenia research (e.g., Calkins and Iacono, 2000; Brownstein et al., 2003; Calkins et al., 2004, 2008; Levy et al., 2004, 2008; Boudet et al., 2005; Heydebrand, 2006), deficits have been identified as schizophrenia spectrum markers (Amador et al., 1991; Sweeney et al., 1994; Faraone et al., 1995; McDowell and Clementz, 1997; Rosenberg et al., 1997; Avila et al., 2002; Kathmann et al., 2003), independent of clinical state (Calkins et al., 2003; Kallimani et al., 2009), or medication (Crawford et al., 1995b; Muller et al., 1999). Furthermore, oculomotor aberrations have been found to fulfill some endophenotypic characteristics (Gottesman and Gould, 2003; Calkins et al., 2008).

Another interesting phenomenon regarding the acquisition of visual information is eye-movement strategies. Eye-movement strategies allow for a meaningful and useful succession of visual information input with regard to, for example, goal attainment or planning behavior (Land and Furneaux, 1997). Deficits in eye-movement strategies may lead to inefficient visual input and may thus alter an individual's perception of the world. As seen in oculomotor deficits, the severity of eye-movement-strategy deficits increases with the increasing progression and severity of schizophrenia. Prodromal individuals have rarely been studied in visual scanning paradigms. Koethe et al. (2006, 2009) showed that abnormal binocular depth inversion was specific to prodromal individuals. Furthermore, first-episode schizophrenia patients have shown an abnormal clustering of fixations and shorter visual scanpaths when scanning faces, landscapes, and abstract patterns (Benson et al., 2007). The largest deteriorations have been reported for stabilized chronic patients with schizophrenia, with fewer fixations, shorter scanpaths, narrower clustering of fixations, and avoidance of predefined features in different visual scanning tasks (e.g., Gaebel et al., 1987; Gordon et al., 1992; Kurachi et al., 1994; Phillips and David, 1997; Williams et al., 1999; Loughland et al., 2002, 2004; Minassian et al., 2005; Koethe et al., 2006).

In our own laboratory, we found that, in contrast to healthy controls, chronic schizophrenia patients do not adapt their eye-movement strategies to task demands but instead use similar strategies regardless of task difficulty (Landgraf et al., 2011b). In other words, patients employ the same visual scanning strategy no matter whether the task is easy or difficult. In another study, we even found that chronic schizophrenia patients use a less efficient information acquisition strategy more often than healthy controls (Landgraf et al., 2011a). This implies that inefficient visual information acquisition impedes patients from obtaining (task-) relevant information. Patients have probably noticed that inflexible visual scanning suits most of the demands they encounter in their daily lives. One intriguing question concerning the discussion of blindness and schizophrenia is whether patients are unable to develop other strategies from birth on or whether patients develop the use of only one strategy despite being able to develop others.

These findings imply the following. First, patients with schizophrenia attribute less weight to visual information and are not able to integrate visual information as well as healthy sighted individuals. This means that patients' deficient performances on higher-order cognitive tasks, therefore, can be accounted for by lower-level visual aberrations. Second, patients' visual channels are still “open.” Hence, they are unable to obtain cognitive and neurofunctional reorganization in the same way as blind individuals. This suggests that PaSZ may be available in two directions. On the one hand, if patients learn to put *more* weight on visual information in the same way that healthy sighted individuals do, they may lower their risk for a psychotic episode. On the other hand, if patients are taught to put *less* weight on visual information and reorganize their neurofunctional information processing, they might be able to stabilize their self and world perceptions in the same way that blind individuals do (See “Vision Training: Decline vs. Improvement” for further details on vision training).

### Visual information processing

Aberrant visual information processing (see “Space” complex in Figure 2) deteriorates thought processes, contributing to cognitive dysfunctions. It has been acknowledged that cognitive deficits may be one of the best predictors of the development of psychosis (Elvevag and Goldberg, 2000; Insel, 2010; Keefe and Harvey, 2012), and this link could provide the basis for quantitative stage markers of the disease. Most of the paradigms used in schizophrenia cognition research are visual tasks. Moreover, fundamental associations between visuo-spatial (VS) abilities and oculomotor capacities (Leigh and Zee, 1999; Hutton et al., 2004; Lawrence et al., 2004; Pierrot-Deseilligny et al., 2005; Milea et al., 2007) indicate that disturbances in the VS domain may stem from oculomotor dysfunctions in patients with schizophrenia. Some of the cognitive domains deficient in patients with schizophrenia include but are not limited to VS memory (Goldman-Rakic, 1994; Green et al., 2000; Piskulic et al., 2007; Landgraf et al., 2011c), VS attention (Posner et al., 1988; Danckert et al., 2004; Granholm and Verney, 2004; Gouzoulis-Mayfrank et al., 2007), and VS executive functions (Laws, 1999; Eisenberg and Berman, 2010; Landgraf et al., 2011a,b,c). An impressive number of studies have actually shown that the development of schizophrenia may be associated with these cognitive deficits and that these cognitive deficits could be stage-specific (Heaton et al., 1994; Niendam et al., 2006; Fusar-Poli et al., 2007; Lysaker et al., 2007; Langdon and Ward, 2008; Picard et al., 2009; Barlati et al., 2012).

Visuo-spatial cognitive deficits in prodromal individuals are controversial. Some studies have shown a greater number of errors and inferior performance in prodromal individuals compared to healthy controls on spatial delayed-response tasks (Wood et al., 2003; Bartok et al., 2005; Smith et al., 2006; Kimhy et al., 2007; Nieman et al., 2007). Other investigations have not confirmed this observation (Brewer et al., 2005; Lencz et al., 2006; Niendam et al., 2006; Pukrop et al., 2007). The reasons for this controversy have not yet been resolved. Possibly, heterogeneous results are due to imprecise diagnoses of the schizophrenia prodrome (high rate of false negatives), demographic differences between participating groups, or task simplicity (Wood et al., 2003; Conklin et al., 2005; Longevialle-Henin et al., 2005; Fusar-Poli et al., 2007; Pukrop and Klosterkotter, 2010). Nevertheless, prodromal individuals have been consistently reported to display deficient performances on the Trail Making Tests (TMT) A and B and the Wechsler Memory Scale (WMS-R) visual reproduction task parts I and II (Hawkins et al., 2004; Brewer et al., 2005).

First-episode patients have shown deficient performances on a number of VS cognitive tasks. For example, they have shown aberrant memory on VS delayed-response tasks (Goldman-Rakic, 1994; Park et al., 1995; Rybakowski and Borkowska, 2002; Simon et al., 2007), Gestalt perception (Parnas et al., 2001), and the TMT (Rybakowski and Borkowska, 2002; Simon et al., 2007). This implies moderate deficits in these patients.

Chronic patients have shown the strongest aberrations on VS tasks. Their deficits include abnormal performance on delayed-response tasks (Park and Holzman, 1992; Goldman-Rakic, 1994; Glahn et al., 2003; Park et al., 2003; Saperstein et al., 2006; Genderson et al., 2007), figure search (Longevialle-Henin et al., 2005), mental rotation (de Vignemont et al., 2006; Halari et al., 2006), Gestalt perception (O'Donnell et al., 1996; Parnas et al., 2001; Cavezian et al., 2007; Kimhy et al., 2007), spatial span (Cannon et al., 2000; Perry et al., 2001; Manoach et al., 2005; Genderson et al., 2007; Thoma et al., 2007), 3-D real-world navigation Daniel et al. (2007), and referencing (Landgraf et al., 2010a; Mazhari et al., 2010). According to a meta-analysis, there is an overall effect size of −1.00 regarding VS working memory deficits in chronic schizophrenia patients (Piskulic et al., 2007), and these deficits are independent of gender differences (Albus et al., 1997; Reichenberg et al., 2002; Voglmaier et al., 2005; Halari et al., 2006; Wolitzky et al., 2006). Hence, visual information processing appears to be deteriorated in schizophrenia, increases with the progression of the disease, and is related to basic visual acquisition. It may be interesting to compare patients' performance to the performance of the blind in non-visual versions of these paradigms. Even though they have never been able to integrate visual information, blind individuals should outperform patients with schizophrenia on spatial tasks. This would indicate that schizophrenia patients' severely altered visual information processing is strongly associated with their deficits in visual information acquisition. We hypothesize that patients with schizophrenia could especially profit from the neurofunctional and cognitive reorganization observed in blind individuals. The most important question, however, for the consideration of PaSZ is whether or not VS deficits are associated with higher-order cognitive dysfunctions and the symptoms of schizophrenia.

### From visual deterioration to symptoms

Basic visual acquisition and processing dysfunctions in patients with schizophrenia suggest a relation to phenotypic symptomatology. In other words, someone who is not able to obtain accurate and precise (visual) information cannot process this information adequately and thus may suffer from deficient amodal representations of the world. Scientifically, visual scanning deficits and oculomotor deteriorations are related to higher-order cognitive dysfunctions (e.g., theory of mind, perspective taking) and social cognition (Adolphs, 2003; Amodio and Frith, 2006; Kluwe-Schiavon et al., 2013), as well as to functional outcomes in schizophrenia (Green et al., 2000; Benson et al., 2007). In fact, the temporo-parietal junction (TPJ), involved in perspective taking and theory of mind, and the insular cortex, involved in body-related multisensory integrations (Arzy et al., 2006; Cavanna and Trimble, 2006; Danckert and Ferber, 2006; Schwabe and Blanke, 2007; Mitchell, 2008), have been associated with psychotic states (Penfield, 1955; Blanke et al., 2005; Vercammen et al., 2010), and impaired whole body and body-part processing in patients with schizophrenia (Tan et al., 2006; Butler et al., 2008b; Suchan, 2008). It has been hypothesized that the failure to predict the sensory consequences of motor commands (Frith et al., 1992, 2000a,b; Friston and Frith, 1995; Blakemore et al., 2002; Frith, 2005) and the improper planning of motor sequences (Delevoye-Turrell et al., 2003; Coello and Delevoye-Turrell, 2007; Voss et al., 2010; Waters and Badcock, 2010) are both essential to schizophrenia symptoms. Furthermore, motor-related deficits in chronic and first-episode patients with schizophrenia include an altered pattern of self-recognition, for example, in the rubber hand illusion (Franck et al., 2001; Versmissen et al., 2007), the inability to distinguish between the self and others (Schwabe and Blanke, 2007; Ebisch et al., 2012), and the identification of the source of self- or externally generated movements (de Vignemont et al., 2006). This implies, on the one hand, that symptom-related deficits in schizophrenia encompass a cognitive component (Bowins, 2011). On the other hand, these results point toward the critical role of multisensory integration deficits in patients (Friston and Frith, 1995; Fourneret et al., 2002) (see “Body” complex in Figure 2).

Because multisensoriality and amodality, as well as self and world representations have already been discussed for blind individuals, there are two things to note. First, the neural sites for multisensory integration may differ between patients with schizophrenia and blind individuals. This would mean that dissimilar neurofunctional processes are conducted. These processes and sites may represent good candidates for early detection markers and possible interventions for schizophrenia. Second, blind individuals and patients with schizophrenia have something in common regarding multisensory integration: they assign less weight to visual information. Blind individuals do so due to the absence of visual input; patients with schizophrenia may do so because they have deficits in visual information acquisition and processing. However, the underlying neurofunctional processes differ and may be an indication of the protective mechanisms of blindness. Interestingly, there is a strong association between oculomotor function and multisensory integration (for reviews, see, e.g., Previc, 1998; Milner and Goodale, 2006). Dysfunctions of visual information acquisition and processing have actually been found to be correlated with multisensory integration deficits (Park and Holzman, 1993; Ross et al., 1998; Jansen et al., 2002; Nieman et al., 2007; Picard et al., 2009) and symptomatology (Gaebel et al., 1987; Lencz et al., 2003; Semerari et al., 2003; Varga et al., 2007) in schizophrenia patients. Moreover, whereas cross-modal influences are dominated by visual information in patients (de Gelder et al., 2005), there is strong evidence that multisensory integration is compromised in chronic patients (Vrtunski et al., 1993; Marvel et al., 2004; Picard et al., 2008; Van den Stock et al., 2011; Castagna et al., 2012). Multisensory facilitation that is established potentially to compensate for deficient unisensory processing in patients with schizophrenia (Javitt, 2009; Williams et al., 2010; Stone et al., 2011) may need to be altered with regard to its reliance on visual information processing (de Gelder et al., 2005). Subsequently, patients might benefit from similar cognitive and perceptual protection against the disease as observed in blind individuals. The degree to which the neglect of visual information integration overlaps between blind and schizophrenic patients in multisensory integration may be an indicator of the severity of schizophrenia. We hypothesize that a greater degree of overlap would indicate a less severe schizophrenia outcome.

## The therapeutic perspective on schizophrenia

### Continuous diagnostic criteria

In the previous section, we demonstrated that deteriorations in the acquisition and processing of visual information increase the risk for developing schizophrenia. We established a critical relation between lower- as well as higher-order visual deteriorations and symptom expression in schizophrenia. Whereas fundamental oculomotor and strategic eye-movement deficits may impact the *acquisition* of visual information, information *processing* deficits point toward VS cognitive aberrations. Both visual information acquisition and processing dysfunctions have been found to increase in severity with the progression of the disorder and are correlated with symptomatic expressions of the disease.

The PaSZ model postulates a continuous relation between visual capacity and the risk of developing schizophrenia. This means that looking at disturbances in vision from a disease progression perspective obviates the need for symptom-based prodromal criteria. Instead, because cognitive deficits can be depicted on much more fine-grained continua than psychotic symptoms (Saperstein et al., 2006; Uhlhaas and Mishara, 2007), we argue that a graded stage model of schizophrenia based on visual functioning will contribute to more reliable diagnostic and especially prodromal criteria for schizophrenia. More importantly, however, are the therapeutic implications of the model, which we will discuss in this final section of the review.

### Vision training: decline VS. improvement

So far, we have presented evidence for why the relative risk of developing schizophrenia is allegedly zero for congenitally blind individuals and for individuals with supernormal visual capacities. According to the PaSZ model, the risk of developing schizophrenia increases from both ends of the visual capacity continuum (congenital blindness and “supernormal” vision) toward a “peak risk” (Figure 1). Thus, depending on the person's initial visual capacity, a decrease in visual impairment or an increase in visual deterioration may similarly *elevate* the risk of developing schizophrenia. Consequently, therapeutic efforts may be differentially effective: a *decline* in a person's visual capacity (increase of visual impairment) may be more beneficial for an individual with visual impairment who never had “normal” visual skills in the first place. By contrast, an *improvement* in a person's visual capacity (decrease of visual deterioration) may be more beneficial for an individual with visual deterioration, that is, who had at some point developed “normal” vision. However, the difference between those two therapeutic approaches may not be clear cut as there is, until now, no clear agreement about (1) how much aberrations of visual functioning corresponds to the highest risk for schizophrenia and (2) whether or not an increase in visual impairment (e.g., via sensory substitution) actually corresponds to a decline in visual functioning.

Nevertheless, vision improvement training is indispensable for PaSZ when the affected individual suffers from visual deterioration. Regarding information *acquisition* (see “Acquisition of visual information” in Table 2), patients should be visually trained to obtain the necessary task-relevant visual information and should be given the tools needed to interpret this information. This means that it may be beneficial to train individuals to utilize task-specific and successful eye-movement strategies. Patients should be taught to direct their attention (eye movements) and cognitive resources (pupil dilation, neurofunctionality) to task-relevant visual information: to look where the information is, to avoid information overload, and to access the “big picture” instead of focusing on attention-captivating details (Johnson et al., 2005; Longevialle-Henin et al., 2005; Cavezian et al., 2007; Coleman et al., 2009; Landgraf et al., 2011b). In addition, training should establish a link between how much weight is given to visual and non-visual information in multisensory integration tasks. Patients should learn when it is advantageous to rely on visual information (fast, parallel processing) and when it is advantageous to rely on non-visual information (slow, sequential processing). This may help them to build more reliable amodal representations of the world, to orient themselves better, and to avoid confusion.

**Table 2:** **“Protection-against-schizophrenia” (PaSZ) – contributions from visual information acquisition and processing**.

**Protective mechanism**	**Functional aspects**	**Training options for schizophrenia patients**
Acquisition of visual information	Scanning for task-relevant information	Directing cognitive resources to the target
		Avoiding information overload
		Global information processing
	Weighing of information	Learning when to rely on which type of information: Visual information = fast, parallel Non-visual information = slow, sequential
Processing of visual information	Neurofunctional sites	Avoiding brain regions associated with symptoms
		Hallucinations, delusions
	Sensory substitution	Learning how to decode visual and spatial information about the environment from non-visual cues

Interestingly, visual deterioration has been associated with the risk for criminal behavior (Bachara and Zaba, 1978; Zinkus and Gottlieb, 1978; Lane, 1980; McKay and Brumback, 1980; Broder et al., 1981; Clack, 1990), one of the strongest indicators of a severe course (Steinert, 1998; Nedopil, 2007; Hutton et al., 2012), outcome (Leygraf, 1988; Haller et al., 2001; Soyka and Morhart-Klute, 2002; Soyka et al., 2002; Fazel et al., 2009; Nitschke et al., 2011; Kooyman et al., 2012), and relapse (Soyka et al., 2004; Witt et al., 2013) in schizophrenia. Specifically, it was argued that in very young children and juveniles, perceptual deficits lead to a higher rate of learning disabilities, specifically reading problems. These problems, in turn, may exclude children from further participating in social interactions and, subsequently, could lead to frustration and feelings of exclusion. It was concluded that visual deterioration in children facilitates the development of delinquent and criminal behavior (Slaton and Jorgensen, 1958; Dzik, 1966, 1975). Vision training was established to counteract this problem and, in fact, it has been found to be effective for reducing criminal behavior and recidivism (Berman, 1989). Because (i) schizophrenia is strongly associated with criminal behavior AND with visual deterioration and (ii) vision training may reduce criminal behavior, the implementation of vision training may be effective for reducing outcome severity in schizophrenia.

Vision training regarding information processing (see “Processing of visual information” in Table 2) may be achieved by functional reorganization. Patients can be taught to utilize different neurofunctional pathways via neurofeedback. Neurofeedback from functional real-time MRI can be used to regulate one's own brain activity, as has been shown predominantly for affective disorders (Linden et al., 2012; Micoulaud-Franchi et al., 2012) but also for the dopamine system (Sulzer et al., 2013) and in Tourette's syndrome (Messerotti Benvenuti et al., 2011). Patients with schizophrenia may learn to avoid certain brain structures associated with hallucinative experiences, such as the Insula and the TPJ. Instead, activation patterns from blind individuals could be mimicked, that is, patients could be taught to activate the occipital lobe for non-visual information processing. Moreover, lower- *and* also higher-order visual information processing may be a target for neurofeedback training in patients with schizophrenia.

Producing declines in a person's visual capacity may be a more radical but also a more effective intervention method especially when the affected individual is suffering from visual impairment. An interesting approach here entails sensory substitution devices for patients with schizophrenia. Intriguingly, vision-deprived individuals can learn to behave in a manner similar to sighted individuals, that is, they may perceive depth, localization, and distance information in real-time from non-visual cues. In a series of inspiring reports, Amedi and colleagues as well as other research groups have shown that visual information can be transmitted via non-visual cues, thus allowing blind individuals to perceive and construct a 3-D image of their environment. The vision-deprived individual obtains VS information auditorily, that is, s/he *hears* visual information. Studies have shown that this type of sensory substitution is easily learned by blindfolded and blind individuals (Amedi et al., 2007; Collignon et al., 2007; Reich et al., 2012). With regard to the etiological considerations of schizophrenia, one interesting research question would be whether or not teaching blind individuals “to see” with sensory substitution devices could circumvent the protective mechanisms of congenital blindness against schizophrenia. This means that if individuals who have acquired the ability to decode VS information from non-visual (e.g., auditory) cues did not develop schizophrenia, cortical reorganization would contribute significantly to blind individuals' immunity against psychosis. However, if this was not the case, modality-unspecific world representations would be more crucial. Sensory substitution devices could then be used for patients with schizophrenia to induce neurofunctional compensation and reorganization for impaired (and possibly deteriorated) visual capacities, accordingly.

The fact that functional reorganization due to sensory deprivation is age-specific is important for all vision trainings (Cohen et al., 1999; Sathian, 2005; Ofan and Zohary, 2007; Cattaneo et al., 2008). For example, independent of sensory input or visual experience, certain brain areas maintain stimulus selectivity (Striem-Amit et al., 2012b), indicating that the human brain has intrinsic constraints with regard to functional plasticity (Striem-Amit et al., 2012d). These constraints must be taken into account when implementing vision training programs.

Finally, visual capacity and schizophrenia show gender effects. Interestingly, disease-related cognitive deficits are not different for men and women, implying that VS capacities do not affect patients' cognitive deficits (Albus et al., 1997; Halari et al., 2006; Wolitzky et al., 2006; Landgraf et al., 2010a). Nevertheless, there are some distinctions regarding the clinical trajectories of men and women who suffer from schizophrenia. Women are diagnosed at an average age of 26.5 years, whereas men are diagnosed earlier at around 21.4 years (Markowitch, 1997). Furthermore, comorbid substance abuse is more frequent in men than in women (Ochoa et al., 2012). Only 7% of female patients with schizophrenia compared to 22% of male patients are convicted of a crime after being discharged from the forensic facility (Soyka et al., 2004). Demo-graphically, 55% of all female forensic patients are single, 15% are married, and 25% are divorced (Melzer, 2001). By contrast, the vast majority of male forensic patients are single (85%), and only 15% are married or divorced (Leygraf, 1988; Nowara, 1993). Suicide rates differ between male and female patients, and men more often successfully commit suicide than women (Markowitch, 1997). When researchers have examined the continuity perspective of schizophrenia, they have rarely taken these gender differences into consideration.

## Conclusion

The ability to see appears necessary but not sufficient for schizophrenia symptoms to develop. The PaSZ model provides a *continuous* measure for assessing the risk of schizophrenia. It suggests that, first, “absent” and “perfect” vision are associated with a lower risk of developing the disorder. Second, there is a peak in schizophrenia risk where visual capacity disturbances are “ideal” for the development of psychosis. Third, both declines AND improvements in visual functioning may improve PaSZ depending on the visual capacities (impaired, deteriorated) of the affected individuals. We argue that the understanding of the causes of schizophrenia and its development can be derived from a continuous vision-based model. And in this review, we presented evidence for this point of view from the “blindness” and the “vision” perspective, ultimately deriving interventional recommendations.

In the “blindness” part, we provided clues about what protects visually impaired, that is, congenitally blind individuals from psychosis. While blind individuals have cognitive experiences that are similar to sighted individuals, they show alterations in cognition (attentional capacities, inhibition of task-irrelevant stimuli, serial processing, strategic adaptation), neurofunctioning (amodal representations, information processing reorganization), and multisensory integration (interference, lateralization, temporal integration, imagery). In fact, these considerations raise the question of how much visual information processing is actually necessary for a person to become vulnerable to psychosis. Or, in other words, how little visual impairment is still protective against manifesting psychotic episodes. Future studies should investigate this and other questions regarding candidates for schizophrenia-specific developmental trajectories. In line with these assumptions, we already hypothesized in a former work that tasks that tap into multisensory integration and full-body motor and navigational control may improve discrimination rates between different disease stages (Landgraf et al., 2012). Nevertheless, individual differences may also contribute to qualitative and quantitative alterations in cognitive functioning regarding blind, vision impaired, and sighted schizophrenic patients (Heller and Kennedy, 1990; Cornoldi et al., 1991; Andreasen and Black, 2006; Andreasen, 2007; Cattaneo et al., 2008).

In the “vision” part, we demonstrated that deteriorated vision, in the form of disturbed visual information acquisition and processing, can lead to the development of schizophrenia symptoms. Specifically, we showed that basic functions, such as oculomotor control and strategic eye movements, are disturbed. Moreover, visuo-cognitive aberrations appear to be based on these deficits, thus resulting in a pattern that leads to motor and self-perception disturbances. These considerations allowed us to pinpoint the etiological underpinnings of schizophrenia because they show that deteriorated visual information acquisition and processing may contribute to the establishment of higher-order cognitive dysfunctions and subsequent symptoms. From a developmental point of view it could be argued that visual capacities may never develop normally in individuals with schizophrenia. If this was the case, the PaSZ model predicts that the majority (if not all) of patients with schizophrenia would be found on the “visual impairment” rather than the “visual deterioration” side. In addition, future research should identify vision- and cognition-related disease stages and determine the time point at which (or the time period in which) interventions may be most important and most effective.

In the “therapeutic” part, we proposed interventional strategies that resulted from the “blindness” and the “vision” perspectives. Decisively, declines in visual capacity (impairment) *and* improvements in visual capacity (deterioration) may increase PaSZ. We formulated treatment options, including vision training, with regard to visual information acquisition (scanning for task-relevant information, weighing information) and processing (neurofunctional sites, sensory substitution). In fact, cortical functional reorganization appears to be most crucial for successful interventions and may be induced by neurofeedback or sensory substitution. Hence, whereas patients would undoubtedly profit from being trained to improve and put *more* weight on visual functioning, we argue that learning how to decline and put *less* weight on visual functioning – the way (congenitally) blind individuals do – may lead to an even stronger protection against psychosis.

In this context, it has to be kept in mind that not all patients suffering from schizophrenia show visual dysfunctions and not all individuals with visual dysfunctions develop schizophrenia. On the one hand, this could be due to the fact that the PaSZ model may only apply to a specific subgroup of patients/individuals. For example, the influence of monocular vision, neurofunctional compensatory mechanisms, and genetic effects need to be investigated in the future. Further, it would be interesting to characterize specific subgroups along etiological dimensions. On the other hand, however, this may also indicate that the current diagnostic (and prodromal) criteria for schizophrenia are too coarse in order to dissociate between different etiological factors. Hence the PaSZ model may provide a fine-grained tool that assesses psychotic symptomatology on the basis of developmental trajectories in much greater detail than current diagnostic procedures. This would result in greater diagnostic precision and, in turn, better therapeutic assignments for affected individuals.

Overall, this review stresses that although the ability to see makes us human (Hegel, 1807; Darwin, 1859; Plato, 380 BC; Wittgenstein, 1921), it also precludes our “PaSZ.” In his “Metaphor of the Sun,” the great Greek philosopher Plato lets his teacher, Socrates, argue that the most important of all senses, *vision*, determines our ways of thinking and how we experience the world (Halfwassen, 2006). Hence, our task of understanding the contribution of the visual system to psychosis will not only eventually lead us to be better able to predict, diagnose, and heal one of the most devastating mental disorders. It will also increase our understanding of how visual functioning influences our ways of thinking and, therefore, our mere existence as human beings.

### Conflict of interest statement

The authors declare that the research was conducted in the absence of any commercial or financial relationships that could be construed as a potential conflict of interest.

## Footnotes

^1^For intelligibility, we use the term “prodromal” to refer to prodromal, ultra-high risk (UHR), or other patients assumed to experience signs of an impending psychotic episode.

^2^Substantial overlap exists between the different approaches (Simon et al., 2006).
